# LUHMES Cells: Phenotype Refinement and Development of an MPP^+^-Based Test System for Screening Antiparkinsonian Drugs

**DOI:** 10.3390/ijms24010733

**Published:** 2023-01-01

**Authors:** Sergei V. Beliakov, Victor Blokhin, Sergey A. Surkov, Michael V. Ugrumov

**Affiliations:** Laboratory of Neural and Neuroendocrine Regulations, Koltzov Institute of Developmental Biology of the Russian Academy of Sciences, 119334 Moscow, Russia

**Keywords:** LUHMES cells, cell culture, dopaminergic neurons, dopamine, 1-methyl-4-phenylpyridinium ion, Parkinson’s disease, neurodegeneration, high performance liquid chromatography, immunocytochemistry, PCR-real time

## Abstract

The low effectiveness of symptomatic pharmacotherapy for Parkinson’s disease (PD), which compensates for dopamine (DA) deficiency under degeneration of nigrostriatal dopaminergic (DAergic) neurons, could apparently be improved with neuroprotective therapy, which slows down neurodegeneration and PD progression. For this, it is necessary to have a DAergic cell line for the development of a PD model to screen neuroprotectors. We used immortalized human embryonic mesencephalon LUHMES cells (LCs) differentiated into DAergic neurons. The aim of this study was to characterize the phenotype of differentiated LCs and develop an 1-methyl-4-phenylpyridinium iodide (MPP^+^)-based test system for screening neuroprotectors. Using polymerase chain reaction (PCR) and immunocytochemistry, it has been shown that all differentiated LCs express genes and synthesize proteins characteristic of all neurons (microtubule-associated protein 2, bIII-tubulin, synaptotagmin 1) and specifically of DAergic neurons (tyrosine hydroxylase, aromatic L-amino acid decarboxylase, DA transporter, vesicular monoamine transporter 2). Furthermore, LCs are able to produce a small amount of DA, but under special conditions. To assess the mechanisms of neurodegeneration and neuroplasticity under the influence of toxins and antiparkinsonian drugs, including neuroprotectors, we have developed an LCs-based MPP^+^ PD model and proposed an original panel of markers for testing functional and structural cell disorders.

## 1. Introduction

Parkinson’s disease (PD) is the second most common neurodegenerative disease after Alzheimer’s disease, characterized by progressive motor disorders, which leads to disability [1]. The key link in the pathogenesis of PD is the selective degeneration of dopaminergic (DAergic) neurons of the nigrostriatal system of the brain, which is involved in the control of motor function [2,3]. These neurons die under the influence of exogenous and endogenous toxic factors. The former includes such environmental factors as pesticides, heavy metals and heroin. Some of them have a selective effect on DAergic neurons due to their capture from the intercellular environment by the dopamine (DA) transporter [4]. The second group of toxic factors is represented by pathologically altered endogenous proteins, which in their native form are necessary for the normal functioning of neurons. In PD, these proteins include α-synucleins–oligomeric and ser-129-phosphorylated, derived from native monomeric α-synuclein, which is involved in DA neurotransmission [5,6].

Despite the fact that the death of nigrostriatal DAergic neurons in PD patients has long been discovered, there is still no effective treatment for this disease, apparently for two reasons [7]. The first reason is that PD, as any other chronic disease, develops for many years without showing the clinical (motor) symptoms that are used to diagnose it. Indeed, PD is first diagnosed and treated only 20–30 years after the onset of neurodegeneration. By this time, 50–60% of DAergic neurons localized in the substantia nigra die, and the level of DA in the axons of these neurons in the striatum decreases by 70–80% [8]. The second reason is that the current symptomatic therapy with DA agonists is aimed at compensating for the deficiency of DA under the degeneration of nigrostriatal DAergic neurons, and not at protecting these neurons from death.

From the above data on the pathogenesis, diagnosis and treatment of PD, it follows that the main goal facing neurologists, neurophysiologists and neuropharmacologists is the development of early (preclinical) diagnosis and preventive neuroprotective therapy of PD. It is believed that effective neuroprotective therapy at the preclinical stage of PD will significantly prolong the asymptomatic development of PD, and, hence, the period of normal social and physical activity of potential patients. In addition, neuroprotective therapy of PD at the clinical stage, apparently, will increase the effectiveness of routine symptomatic therapy [9]. To do this, it is necessary to ensure the screening of a large number of already known and newly produced synthetic and plant-derived drugs with neuroprotective properties [10]. Before clinical trials, the neuroprotective and side effects of these substances should be thoroughly characterized, first in in vitro models and then in animal models of PD. In this context, the greatest burden falls on in vitro models, as the first stage of preclinical trials.

Until the last decade, cell lines of neuroblastoma (SH-SY5Y, SK-N-BE) of human and animal origin, as well as pheochromocytoma (PC12) of animal origin, were widely used to model PD [11,12,13,14,15,16,17]. A common serious disadvantage of these cells is that they express a mixed phenotype. Along with functionally significant proteins of DAergic neurons, they express proteins characteristic of noradrenergic, cholinergic, and a number of other non-DAergic neurons [13,18,19,20]. The primary culture of rodent mesencephalon is also of little use for modeling PD [21,22], whereas obtaining human embryonic mesencephalon (abortion material) is not available for ethical reasons. Although the recently developed method for obtaining DAergic neurons from stem cells of PD patients is promising for the development of the mentioned cell test system, this approach is too time-consuming and expensive for its regular use [23,24,25,26].

One of the most promising, if not the most promising, cell model for screening of neuroprotectors for DAergic neurons of the substantia nigra are LUHMES cells (LCs), which are immortalized human DAergic neurons [27,28,29]. The advantages of these cells are that: (i) they are derived from human mesencephalon; (ii) they easily and quickly reproduce and differentiate into DAergic neurons, and these procedures are not expensive; (iii) they are suitable for modeling PD using the neurotoxin of DAergic neurons such as 1-methyl-4-phenylpyridinium iodide (MPP^+^), rotenone and pesticides [30,31]. A comparative analysis of the main subpopulations of LCs, UKN (University of Konstanz, original provider) and the American Type Culture Collection (ATCC), showed that the differentiated cells of both cell lines express DAergic phenotype, although with minor differences in the degree of the expression of some genes and proteins. Differences observed between subpopulations of LCs are explained by “genetic drift” due to uncontrolled cell proliferation [32]. It should be noted that the phenomenon of “genetic drift” is also characteristic of other cell lines [33]. It follows from these data that LCs can be used for drug screening by any team, but only after careful analysis of the phenotype of each cell subpopulation used. This obligatory study is also useful for general clarification of their neuronal and especially DAergic phenotype. The aim of this study was to characterize the phenotype of the LC subpopulation at our disposal and to develop an MPP^+^-based test system for screening antiparkinsonian drugs.

## 2. Results

### 2.1. Specification of Differentiated LUHMES Cells

#### 2.1.1. Expression of Neuronal Phenotype Proteins

**Figure 1:**
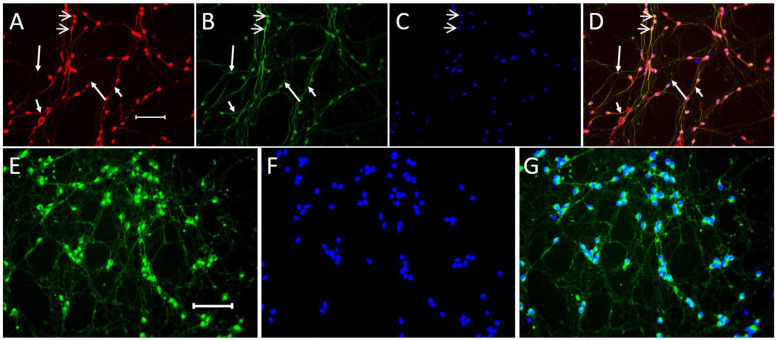
Neuronal marker proteins detected immunocytochemically in differentiated LUHMES cells. (**A**,**B**,**D**): Double-immunolabeling for microtubule-associated protein 2 (MAP2) ((**A**), red) and β-tubulin III ((**B**), green) and their colocalization ((**D**); yellow). (**E**,**G**): synaptotagmin 1 monoimmunolabeling (green). Cell nuclei are stained with 4′,6-diamidino-2-phenylindole dihydrochloride (DAPI) ((**C**,**F**), blue). Open arrows, double immunolabeled neurons; long arrows, neuron processes monoimmunolabeled for β-tubulin III; short arrows, neuron processes with double immunolabeling for β-tubulin III and MAP2. Bar: 100 μm.

On the 7th day of differentiation of LCs, their neuronal phenotype was immunocytochemically tested, identifying the following neuronal markers: β-Tubulin III, a marker of a neuron distributed along its entire length, and microtubule-associated protein 2 (MAP2), a marker of cell bodies and mainly processes of neurons (neurites), as well as synaptotagmin 1, a presynaptic protein involved in the transport of neurotransmitters across the membrane. Colocalized β-Tubulin III and MAP2 were detected in differentiated LCs using double immunolabeling. With this approach, it was shown that differentiated LCs also contain synaptotagmin 1-immunopositive material (Figure 1).

#### 2.1.2. Expression of Genes for Proteins of the Dopaminergic Phenotype

**Figure 2:**
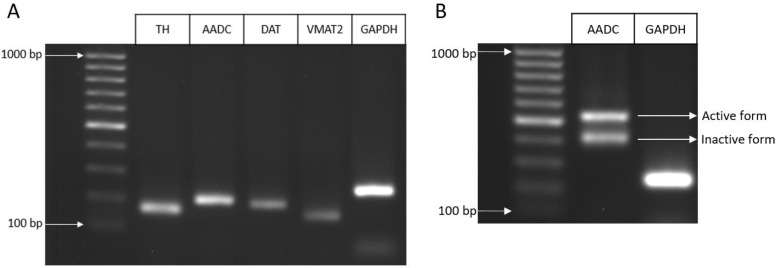
(**A**): expression of dopaminergic phenotype protein genes: tyrosine hydroxylase (TH), dopamine transporter (DAT), aromatic L-amino acid decarboxylase (AADC), vesicular monoamine transporter 2 (VMAT2), as well as the housekeeping gene glyceraldehyde-3-phosphate dehydrogenase (GAPDH); (**B**): agarose phoresis for the AADC mRNA region between exons 2 and 5. Several forms of AADC are visible. The lower band corresponds to the gene for the enzymatically inactive form of AADC, which lacks exon 3, while the upper band corresponds to the gene for the enzymatically active form of AADC.

At the first stage of testing the expression of the DAergic phenotype by LCs, polymerase chain reaction (PCR) of the expression of the tyrosine hydroxylase (TH), DA transporter (DAT), aromatic L-amino acid decarboxylase (AADC), and vesicular monoamine transporter 2 (VMAT2) genes was performed. The expression of all these genes was found (Figure 2A). Changes in gene expression in LCs on day 7 of differentiation compared to undifferentiated cells (normalized for GAPDH) are shown in Figure S1. Considering the literature data on the existence of several AADC forms resulting from alternative splicing [34], we evaluated the possibility of AADC alternative splicing in LCs in the region between exons 2 and 5 using PCR and found several mRNA forms of this enzyme (Figure 2B).

#### 2.1.3. Expression of Dopaminergic Phenotype Proteins

**Figure 3:**
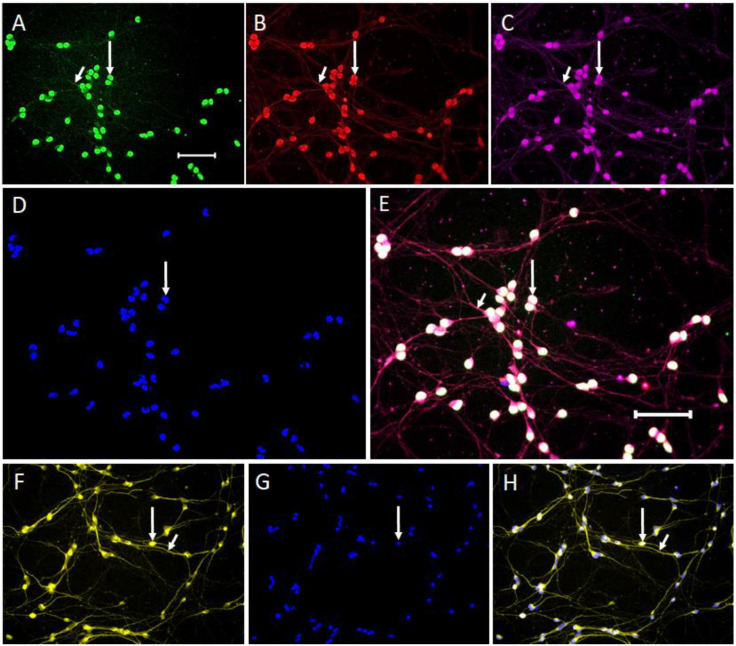
Triple immunolabeling of LUHMES cells for: tyrosine hydroxylase (TH) ((**A**), green), aromatic L-amino acid decarboxylase ((**B**), red), and dopamine transporter ((**C**), magenta), and staining of cell nuclei with 4′,6-diamidino-2-phenylindole dihydrochloride (DAPI) ((**D**), blue). Colocalization of all four markers results in white staining of cells (**E**). Monoimmunolabeling of LUHMES cells for tyrosine hydroxylase with the use of rabbit-anti-TH ((**F**), yellow), staining of cell nuclei with DAPI ((**G**), blue), merged result ((**H**), lilac). Long arrow, cell bodies stained with all four markers; short arrow, cell processes (neurites) immunostained for three proteins of the dopaminergic phenotype. Bar: 100 µm.

The DAergic phenotype of LCs was tested using monoimmunolabeling and triple immunolabeling for the following proteins: TH, DAT, AADC, and VMAT2. All of these proteins were identified in all LCs using monoimmunolabeling. Moreover, using triple immunolabeling for TH, AADC, and DAT, colocalization of these proteins was detected in all LCs (Figure 3A–E). Since TH immunolabeling with monoclonal antibodies (Mouse-anti-TH, Sigma, St. Louis, MO, USA) did not show clearly immunostained neurites in the triple immunolabeling experiment, we additionally performed TH monolabeling using Rabbit-anti-TH, Thermo Fisher, Waltham, MA, USA (Figure 3F–H).

To prove that all differentiated LCs express DAergic phenotype proteins, we compared the number of 4′,6-diamidino-2-phenylindole dihydrochloride (DAPI)-labeled cells with the number of: TH-immunopositive cells (DAPI: 65 ± 9.2 vs. TH: 65 ± 9.2, *p* > 0.05), AADC-immunopositive cells (DAPI: 65 ± 9.2 vs. AADC: 65 ± 9.2, *p* > 0.05), DAT-immunopositive cells (DAPI: 65 ± 9.2 vs. DAT: 65 ± 9.2, *p* > 0.05) per 0.14 mm^2^ of culture. These data show 100% colocalization of DAPI and proteins of the DAergic phenotype. In the immunocytochemical study of undifferentiated LCs, we detected the expression of AADC and VMAT2, but did not detect the expression of TH and DAT (Figure S2).

**Figure 4:**
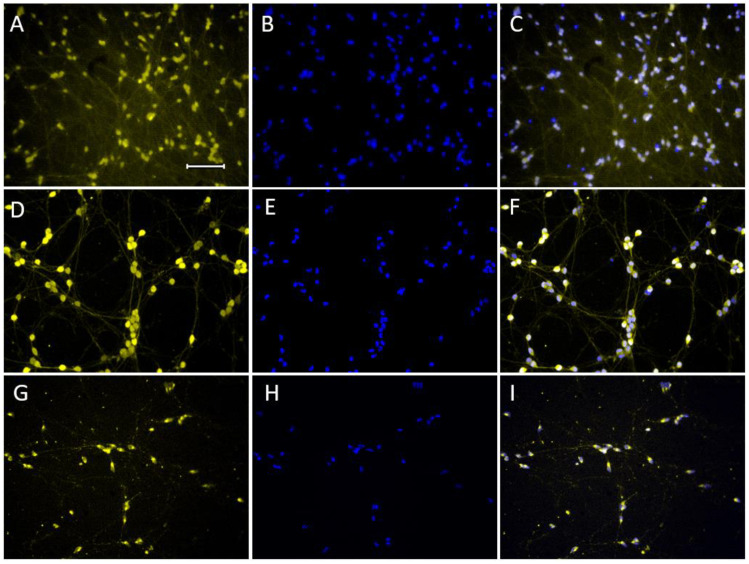
Monoimmunolabeling of LUHMES cells for vesicular monoamine transporter 2 (VMAT2) ((**A**,**C**), yellow) and α-synuclein, monomeric ((**D**,**F**), yellow) and phosphorylated at Ser-129 ((**G**,**I**), yellow). LUHMES cells were additionally stained with 4′,6-diamidino-2-phenylindole dihydrochloride (DAPI) ((**B**,**E**,**H**), blue), a nuclear dye. Bar: 100 µm.

Using monoimmunolabeling, LCs were shown to contain not only TH, AADC, and DAT, but also VMAT2 and α-synuclein, monomeric and phosphorylated at Ser-129 (Figure 4).

#### 2.1.4. Content of L-3,4-dihydroxyphenylalanine, pyridoxal-5-phosphate and Dopamine in LUHMES Cells and in the Incubation Medium under Various Conditions

In the first series of experiments, some cells were incubated for 30 min in artificial cerebrospinal fluid. L-3,4-dihydroxyphenylalanine (L-DOPA) was detected in the medium and in cells, at a concentration of not more than 50 pmol/mg of cell protein and less than 1 pmol/mg of cell protein, respectively. Pyridoxal-5-phosphate was detected in cells at the concentration of 42.6 ± 10.8 pmol/mg of cell protein, and in artificial cerebrospinal fluid at the concentration of 69.5 ± 22.8 pmol/mg of cell protein (Figure S3).

Other cells were incubated in artificial cerebrospinal fluid containing pargyline, a monoamine oxidase inhibitor. As previously, we have detected L-DOPA, but not DA in the incubation medium and cells.

**Figure 5:**
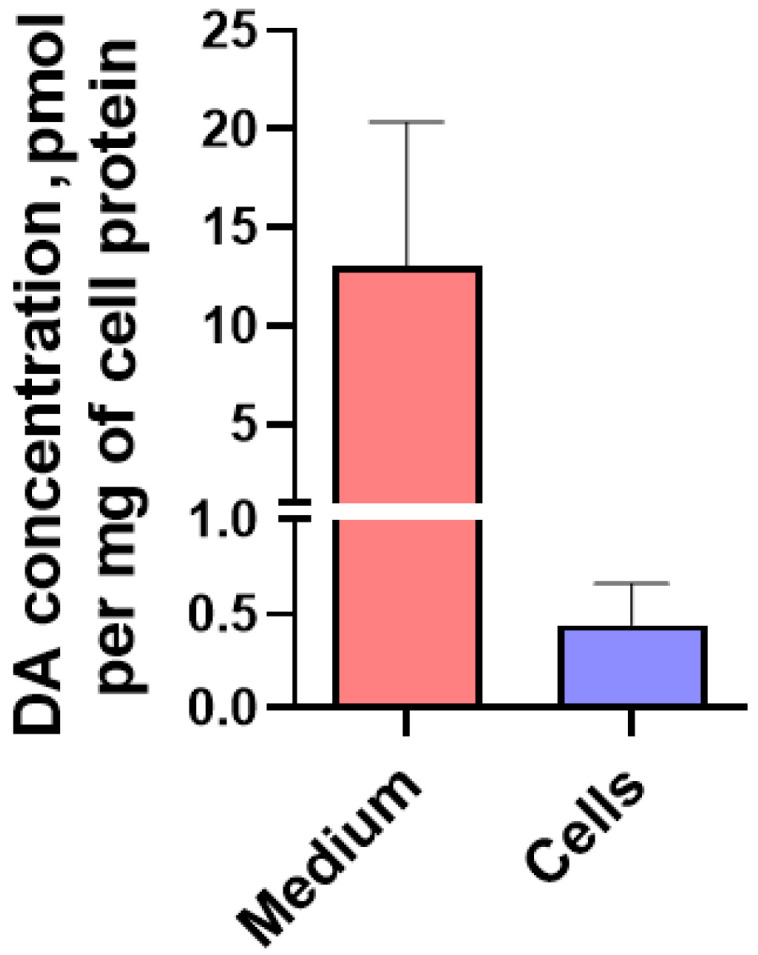
Dopamine (DA) content normalized to cell protein in LUHMES cells and incubation medium (artificial cerebrospinal fluid) containing pargyline (30 µM), tolcapone (10 µM), and L-DOPA (100 nM).

In the second series of experiments, LCs were incubated in artificial cerebrospinal fluid containing pargyline (30 μM) and tolcapone (10 μM) for 30 min, followed by the addition of L-DOPA (100 nM) and incubation for another 1.5 h. In this case, DA was detected both in the medium (13.08 ± 7.30 pmol/mg of protein) and in cells (0.44 ± 0.38 pmol/mg of protein), in the latter to a much lesser extent (Figure 5).

**Figure 6:**
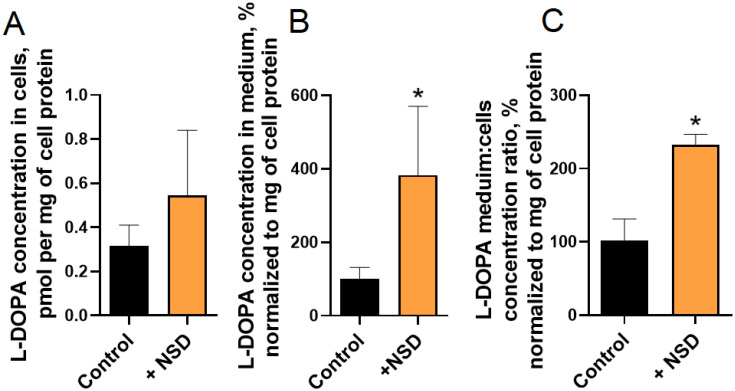
The concentration of L-DOPA in LUHMES cells (**A**), incubation medium (**B**), and their ratio (**C**) following cell incubation in artificial cerebrospinal fluid containing 500 μM of 3-hydroxybenzylhydrazine dihydrochloride (NSD-1015). * *p* < 0.05, significant difference compared to the control.

In the third series of experiments, when 3-hydroxybenzylhydrazine dihydrochloride was added to the medium at a concentration of 500 μM, the concentration of L-DOPA in the cells, normalized to the protein in the cells, did not change, while the concentration of L-DOPA in the medium, normalized to the cell protein, increased significantly (Figure 6).

### 2.2. Modeling Parkinson’s Disease on LUHMES Cells Using 1-methyl-4-phenylpyridinium Iodide (MPP^+^)

PD was modeled on LCs with MPP^+^, a toxin of DAergic neurons that is captured into neurons by DAT [35,36,37].

#### 2.2.1. Evaluation of the Functional Activity of Mitochondria in LUHMES Cells Using the (3-(4,5-dimethylthiazol-2-yl)-2,5-diphenyltetrazolium Bromide (MTT) Test when Exposed to 1-methyl-4-phenylpyridinium Iodide

**Figure 7:**
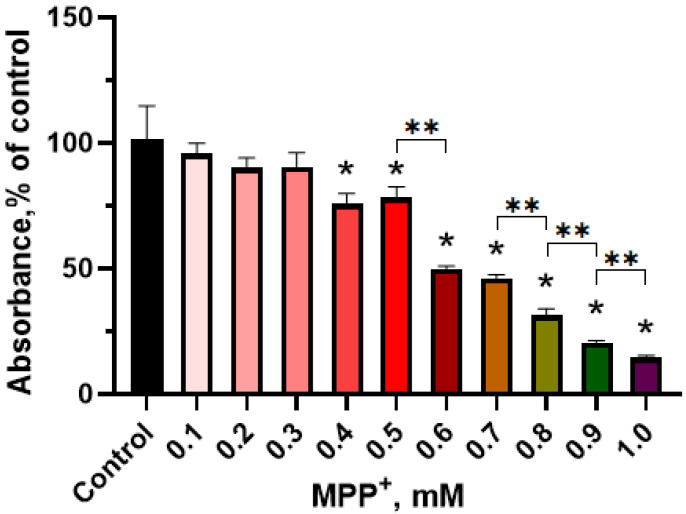
Formazan absorption resulting from 3-(4,5-dimethylthiazol-2-yl)-2,5-diphenyltetrazolium bromide (MTT) reduction in differentiated LUHMES cells after their incubation for 48 h with 1-methyl-4-phenylpyridinium iodide (MPP^+^) at doses from 0.1 up to 1.0 mM. * *p* < 0.05, significant difference compared to the control, ** *p* < 0.05, significant difference between the selected groups.

We have evaluated the dose dependence of the functional activity of mitochondria in differentiated LCs on the concentration of MPP^+^ after incubation of these cells in a differentiation medium with MPP^+^ at a concentration of 0.1 mM to 1.0 mM for 48 h. This indicator was measured on a spectrophotometer as the level of absorption of formazan, a product of 3-(4,5-dimethylthiazol-2-yl)-2,5-diphenyltetrazolium bromide reduction. The first disruption in the functional activity of mitochondria in LCs has been detected at an MPP^+^ concentration of 0.4 mM in the incubation medium. These changes progressed with increasing concentration of MPP^+^ up to the maximum used concentration of 1 mM (Figure 7).

#### 2.2.2. Evaluation of 1-methyl-4-phenylpyridinium Iodide-Induced Degeneration of LUHMES Cells

**Figure 8:**
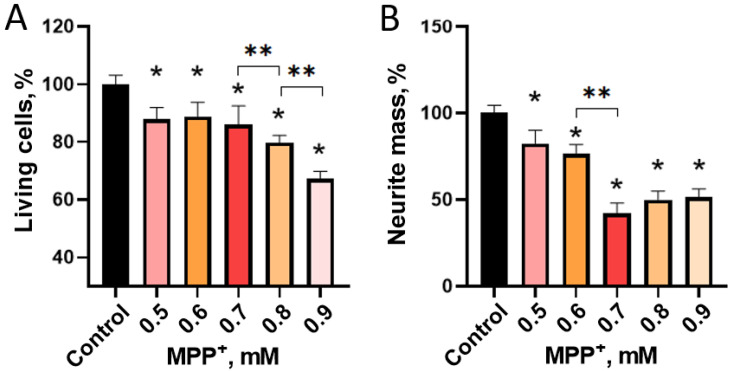
The number of LUHMES cells (**A**) and the mass of neurites (**B**) after their incubation in a differentiation medium with 1-methyl-4-phenylpyridinium iodide (MPP^+^) at a gradually increasing concentration compared to the control (incubation without MPP^+^). * *p* < 0.05, significant difference compared to the control, ** *p* < 0.05, significant difference between the selected groups.

**Figure 9:**
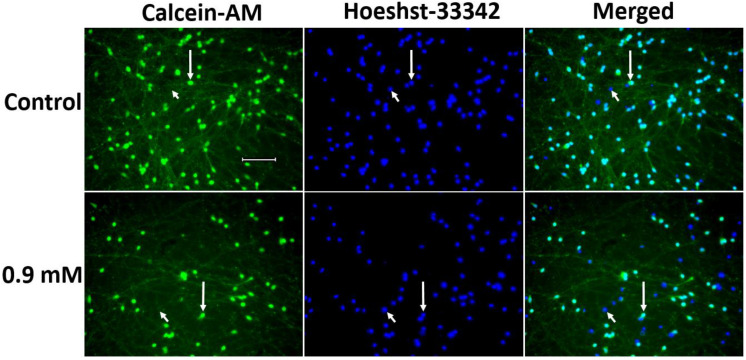
Decrease in the number of cell processes of LUHMES cells (“neurite mass”) and the number of LUHMES cells stained with calcein-AM (calcein-acetoxymethyl ester) (green) after incubation for 48 h with 1-methyl-4-phenylpyridinium iodide at a concentration of 0.9 mM compared to control. Cell nuclei are stained with Hoeshst-33342 (blue). Long arrows, living LUHMES cells; short arrows, dead cells. Bar: 100 µm.

It was shown that the incubation of LCs in the differentiation medium with MPP^+^, starting from a concentration of 0.5 mM, leads to a decrease in the mass of neurites and the number of cell bodies stained with calcein-acetoxymethyl ester (hereinafter—calcein) (Figure 8 and Figure 9). The maximum decrease in the number of LCs was observed upon their incubation with 0.9 mM MPP^+^, and the maximum decrease in the mass of processes occurs upon incubation with 0.7 mM MPP^+^ (Figure 8 and Figure 9).

## 3. Discussion

The lack of effective pharmacotherapy for PD makes it necessary to improve current symptomatic therapy aimed at compensating for the DA deficiency resulting from degeneration of nigrostriatal DAergic neurons [1,9,38]. However, a fundamental breakthrough in the fight against PD, expected in the foreseeable future, will occur with the development of early (preclinical) diagnosis and preventive neuroprotective therapy. This will slow down neurodegeneration, significantly prolonging the preclinical stage of PD, which is the period of comfortable life for the patient [9,39]. Since neurodegeneration is a complex pathological process that includes oxidative stress, neuroinflammation, apoptosis, and toxic effects of α-synuclein derivatives, neuroprotective therapy in PD should be multitargeted [40], using neuroprotectors with different mechanisms of action.

Considering that many neuroprotectors are already known, and the list of neuroprotective candidates is rapidly expanding, it is necessary to improve current and develop new in vitro PD models for screening potential drugs. In this context, the greatest hopes are placed mainly on neurotoxic PD models based on DAergic neurons obtained from the stem cells of PD patients [41] or from immortalized embryonic LCs cells of the human midbrain [27]. To assess the protection of “healthy” DAergic neurons from the action of exogenous and endogenous toxic factors due to neuroprotectors, human DAergic neurons derived from LCs seem to be preferable for a number of reasons. The most important advantage of these cells over other cell lines is the rapid and massive reproduction of DAergic neurons at relatively low cost [29]. However, when using LCs, it should be kept in mind that although both LC subpopulations, ATCC and UKN, express a DAergic phenotype, the degree of expression of some genes and proteins differs slightly (Table 1).

It is assumed that the observed differences between LC subpopulations are explained by “genetic drift” due to uncontrolled cell proliferation [32], which is also characteristic of other cell lines [33].

Based on the above, the first objective of this study was to characterize the phenotype of the LC subpopulation at our disposal on the 7th day of their differentiation. According to most researchers, by this time, LCs pass through the main stages of differentiation [29], which is consistent with our data (see Results and Supplementary Materials). Indeed, on the 7th day of differentiation according to the protocol described in [29], almost all LCs are morphologically similar to neurons, most often bipolar, although multipolar neurons are also visible. They also manifest a neuronal chemical phenotype by expressing two key neuronal marker proteins, MAP2 and βIII-tubulin. Our immunocytochemical study confirmed the idea that bIII-tubulin is contained both in the cell bodies of differentiated LCs and in neurites throughout their entire length. Although the second neuronal marker protein, MAP2, such as βIII-tubulin, is found in the cell bodies and processes of differentiated LCs, it is not present throughout the entire length of neurites and not in all neurites. These data confirm to some extent the notion that MAP2 is localized predominantly in dendrites [45,46]. Finally, on the 7th day of differentiation, we detected with immunocytochemistry synaptotagmin 1 in LCs, in cell bodies, and in neurites. In addition to the neuronal cell bodies, the highest content of synaptotagmin 1-immunopositive material was observed in transient and terminal neurite varicosities. Considering that synaptotagmin 1 is one of the presynaptic proteins of the vesicular cycle [47], our data suggest that the chemical machinery for neurotransmission in LCs is developed by the 7th day of differentiation.

When evaluating the phenotype of differentiated LCs, it is especially important to determine to what extent the LCs at our disposal express the DAergic phenotype and, therefore, can be used to develop a PD cell model as a test system for screening neuroprotectors. At the first stage of this study, we proceeded from three prerequisites: (i) the key marker of DAergic neurons is TH, the first rate-limiting enzyme of DA synthesis; (ii) TH is expressed in LCs starting from the 4th day of cultivation, reaching a plateau by the 7th day [30]; (iii) TH is expressed in LCs only when cultured in a medium containing dibutyryl 3′,5′-cyclic adenosine monophosphate and glial cell line-derived neurotrophic factor [29]. Following these requirements, we found that on day 7 of differentiation, 100% of LCs with DAPI-stained nuclei contained TH-immunopositive material. In the same cell population, we showed TH gene expression by PCR. Surprisingly, Harischandra et al. [42] were able to detect a maximum of 25% of cells expressing TH using the same protocol for LC differentiation. The only idea might be that they used antibodies with low sensitivity to TH.

After the first step of assessing the DAergic phenotype of differentiated LCs in our subpopulation, we tested gene expression and synthesis of a number of functionally important proteins characteristic for DAergic neurons. Thus, on the 7th day of LC differentiation, we detected by PCR the gene expression of the following proteins: AADC, the second enzyme of DA synthesis; VMAT2, a vesicular membrane transporter type 2 that provides DA storage in vesicles; and DAT, a membrane transporter that captures DA from the extracellular space [48]. At the next stage of the study, to assess the expression of functionally significant proteins of the DAergic phenotype in differentiated LCs, we used various immunocytochemical methods: monoimmunolabeling, double immunolabeling, and triple immunolabeling. Comparison of the number of LCs with nuclei stained with DAPI and cells immunostained for DAT or AADC showed that, like TH, DAT and AADC are expressed in all LCs. Of particular interest is our triple immunolabeling data showing the colocalization of TH, AADC, and DAT in all LCs.

Despite the fact that LCs differentiated into DAergic neurons were immunostained for all three neuronal markers throughout the cell, the highest concentration of immunopositive material was observed in the cell bodies and in neurite varicosities, which are most likely “presynaptic terminals”. Additional use of monoimmunolabeling showed that LCs on day 7 of culture also contained VMAT2. Immunostained VMAT2 and synaptotagmin 1 can be considered as markers of the chemical machinery for DA release by exocytosis of DA-storing vesicles.

Interesting information was obtained in our study when comparing the expression of genes and proteins of DAergic phenotype in undifferentiated and differentiated LCs. For example, it has been shown that differentiated LCs, in contrast to undifferentiated cells, have reduced DAT gene expression (qPCR data) and DAT protein is synthesized (immunocytochemistry data). Similar data were obtained by Leist and colleagues in a previous study, but these data were not discussed [32]. In the future, it is desirable to study the regulation of transcription and translation of DAT and some other proteins of the DAergic phenotype in LCs.

Along with specific proteins which are markers of the DAergic phenotype, α-synuclein, native and phosphorylated at Ser-129, was detected in LCs using monoimmunolabeling. Despite the fact that α-synuclein is not a marker protein of DAergic neurons, its metabolism in PD is impaired mainly in DAergic neurons of the nigrostriatal system [49]. Therefore, the detection and assessment of the content of α-synuclein and its pathological toxic derivatives are of great value in the screening of neuroprotectors on cell models of PD.

After analyzing the expression of genes and proteins responsible for the synthesis and release of DA in differentiated LCs, the functioning of the chemical machinery for DA neurotransmission was evaluated. First, it was necessary to determine whether the differentiated LCs at our disposal synthesize DA, as has been previously shown for some other subpopulations of LCs [29,32]. However, in contrast to previous studies [29,32], we failed to detect DA either in differentiated LCs or in the incubation medium, which could be due to the low activity of DA-synthesizing enzymes. This assumption is indirectly supported by Gutbier et al. [32], who showed that TH gene expression in the ATCC subpopulation is 10 times lower than in the UKN subpopulation. However, the direct answer to this question could only be obtained by evaluating the activity of DA-synthesizing enzymes, primarily TH, as a rate-limiting enzyme.

For the first time, we tried to evaluate the activity of TH in LCs by measuring the content of L-DOPA. We succeeded in detecting L-DOPA in differentiated LCs and in the incubation medium and showed that its content increases upon inhibition of AADC by 3-hydroxybenzylhydrazine dihydrochloride. Interestingly, the content of L-DOPA in incubated LCs is about 100 times lower than in the incubation medium—Artificial cerebrospinal fluid. Most likely this is due to the pumping out of L-DOPA from cells via L-type amino acid transporter 1 [50,51].

The above data prove that the TH protein detected by immunostaining is enzymatically active. On the other hand, these data show that although L-DOPA is metabolized by AADC, this does not result in the synthesis of sufficient amount of DA for high performance liquid chromatography (HPLC) detection. We suggested that this may be due to several concerns: rapid degradation of endogenous DA by monoamine oxidase, low enzymatic activity of AADC, or low content of endogenous L-DOPA as a substrate for DA synthesis. When evaluating the metabolism of L-DOPA and DA, it should be taken into account that the lifetime of DA in brain tissue is no more than 2 s [52]. Given these data, we incubated LCs with pargyline, a monoamine oxidase inhibitor, but this did not result in the detection of DA. We then supplemented this experiment by introducing tolcapone, an inhibitor of L-DOPA degradation, and exogenous L-DOPA in a high concentration into the incubation medium. In this case, we were able to detect DA, which may be due to two points. First, AADC, as shown previously, can hypothetically be represented by several isoforms, including those without enzymatic activity, such as AADC442, which lacks the 3rd exon [53,54,55]. Our PCR analysis of LCs has shown for the first time that they express several AADC protein genes, including a defective variant lacking the 3rd exon. This may explain the fact that much less DA is synthesized in LCs than in some other DAergic cell lines. Indeed, according to Scholz [29], in the UKN subpopulation of LCs, 18 ng of DA is synthesized per 1 million differentiated cells, whereas in the ATCC subpopulation, the outcome of DA synthesis is 40% lower. At the same time, a million MN9D cells contain 102 ng of DA [56], and a million DAergic neurons in a primary culture of mouse embryonic midbrain contain 214 ng of DA [57].

The second and more realistic hypothesis is that LCs contain a high concentration of some substances that compete with L-DOPA for AADC. In order to more accurately answer the question about the reasons for the limitations in the conversion of L-DOPA to DA, it is further desirable to estimate the content and enzymatic activity of various isoforms of the AADC protein in LCs, as well as to identify hypothetical endogenous substances that compete with L-DOPA for AADC.

A detailed refinement of the chemical phenotype and functional activity of the LCs at our disposal made it possible to develop a PD model that mimics the gradual degeneration of DAergic neurons, ranging from minor functional and structural (morphological) impairments to cell death. This cell model of PD should be useful for evaluating the effectiveness of neuroprotective candidates at various stages of DAergic neuron degeneration. When developing such a model, we took as a basis the widespread modeling of PD using MPP^+^, a specific toxin of DAergic neurons [58]. Penetrating into DAergic neurons thanks to DAT, MPP^+^ causes uncoupling of oxidative phosphorylation in mitochondria and oxidative stress [59]. The same mechanism of action is characteristic of a number of other exogenous neurotoxins, for example, heroin, which causes the development of PD in humans [39,60].

In our opinion, the low level of DA synthesis in differentiated LCs should not affect the degeneration of these cells under the influence of MPP^+^ in modeling PD. Indeed, the cytotoxic effect of MPP^+^, which manifests itself at the cellular level in the degradation of neurites and cell death, is not accompanied at the subcellular level by a direct effect on the molecules involved in the synthesis of DA–TH, AADC, and cofactors of these enzymes. DA may have a toxic effect on DAergic neurons, but only when it accumulates in the cytoplasm due to increased synthesis and/or decreased uptake into vesicles by VMAT2 [61,62].

In a number of studies devoted to the development of a PD model on LCs using MPP^+^, it is emphasized that the ATCC cells at our disposal are much less sensitive to the toxic effect of MPP^+^ than the UKN cells [10,32,63,64]. This is mainly due to a much lower level (about 50 times) of DAT expression in ATCC cells compared to UKN cells [32]. The degeneration of differentiated LCs is usually assessed by such indicators as: a decrease in ATP synthesis, an increase in caspase activity, a decrease in the intracellular content of glutathione, an increase in the release of lactate dehydrogenase from cells, and an increased formation of malondialdehyde [47].

To assess the degree of damage to LCs under the influence of MPP^+^, we proposed for the first time to use a panel of neurodegeneration markers, which makes it possible to simultaneously assess functional and structural impairments in cells. The 3-(4,5-dimethylthiazol-2-yl)-2,5-diphenyltetrazolium bromide test, which determines the functional activity of mitochondria, was used as an indicator of functional changes in LCs. In particular, this test can be used to determine the critical concentration of MPP^+^ at which it triggers intracellular toxic effects. For the differential assessment of structural changes, we used calcein staining of LCs, followed by computer quantification of neurite loss and cell loss. This approach, together with the 3-(4,5-dimethylthiazol-2-yl)-2,5-diphenyltetrazolium bromide test, provides a detailed characterization of the degradation and survival of a cell culture after exposure to a toxin or a potential neuroprotector. Thus, using both approaches, we have shown that the effect of MPP^+^ on the functional and structural parameters of differentiated LCs is dose-dependent. The first functional changes detected by the 3-(4,5-dimethylthiazol-2-yl)-2,5-diphenyltetrazolium bromide test, and structural changes, specifically, a decrease in the mass of neurites and the number of cell bodies, were noted at the same concentration of MPP^+^—0.5 mM. With an increase in the concentration of MPP^+^ to 0.6–0.7 mM, an abrupt decrease in the functional activity of mitochondria and the mass of neurites was observed, while the number of cell bodies remained practically unchanged compared to the dose of 0.5 mM. With a further increase in the concentration of MPP^+^ from 0.7 to 0.9 mM, an abrupt decrease in the functional activity of mitochondria and a progressive decrease in the number of cell bodies were simultaneously observed. In contrast to the number of cell bodies, the mass of neurites did not change in this range of MPP^+^ concentrations.

Thus, we have obtained evidence that all differentiated LCs (subpopulation of the AATC cell line) at our disposal express neuronal and DAergic phenotypes, while using MPP^+^, we have developed a PD cell model that can be applied as a test system for mass screening of potential neuroprotectors of DAergic neurons.

## 4. Materials and Methods

### 4.1. LUHMES Cell Cultivation

LCs of the CRL-2927 line were obtained from the ATCC.

#### 4.1.1. Preparing Plastic Dishes

Since both undifferentiated and differentiated LCs have adhesive properties, for their successful attachment and cultivation, plastic dishes were treated according to the subculturing procedure described by the manufacturer, ATCC. The working surface of a plastic dish was sequentially pre-coated with a solution of poly-L-ornithine (Sigma) (50 µg/mL) overnight at 20 °C and then with a solution of human plasma fibronectin (Panbiotech, Aidenbach, Germany/Imtek, Moscow, Russia) (1 µg/mL) for 3 h at 37 °C. Before further use, plastic dishes were washed with distilled water and dried under sterile conditions.

#### 4.1.2. Subculturing Undifferentiated Cells

Undifferentiated LCs, obtained from ATCC, were cultured in DMEM/F12 growth medium (1:1) (Gibco, Waltham, MA, USA) supplemented with 1% serum-free N-2 (Gibco), 2 mM Glutamine, 15 mM HEPES (Gibco), and basic fibroblast growth factor (Gibco) (20 ng/mL). When the cells covered about 80% of the area of the Petri dish (60 mm), the culture medium was removed and the cells were washed 3 times with 1.0 mL Dulbecco’s phosphate-buffered saline (DPBS) without Ca^2+^ and Mg^2+^ (Gibco). Before the cells were removed from the substrate, they were incubated in 1.5 mL trypsin solution (0.05%) with EDTA (0.02%) (Sigma) for 5 min at 37 °C. After that, the cells were collected from the dish surface, by washing them off by pipetting. The cell suspension in trypsin solution was placed into a centrifuge tube and 1:3 with DMEM/F12 medium without additives. The resulting solution with cells was centrifuged for 10 min at 200× *g* for 7 min at 20 °C. The supernatant was removed and the precipitate was resuspended by pipetting in 2 mL proliferation medium. After that, the number of cells was counted in a hemocytometer. To maintain the stock culture, the cell suspension in the growth medium was sown into a Petri dish (60 mm diameter), 0.5–1.0 × 10^6^ cells per dish (density = 2.4–4.8 × 10^4^/cm^2^). Cultivation was carried out in an incubator (SANYO, Osaka, Japan) at 37 °C in humidified air and 5% CO_2_. The medium was changed every 2 days. The culture was reseeded every 3–4 days. The experiments were carried out after the second passage.

#### 4.1.3. Cell Differentiation

**Figure 10:**
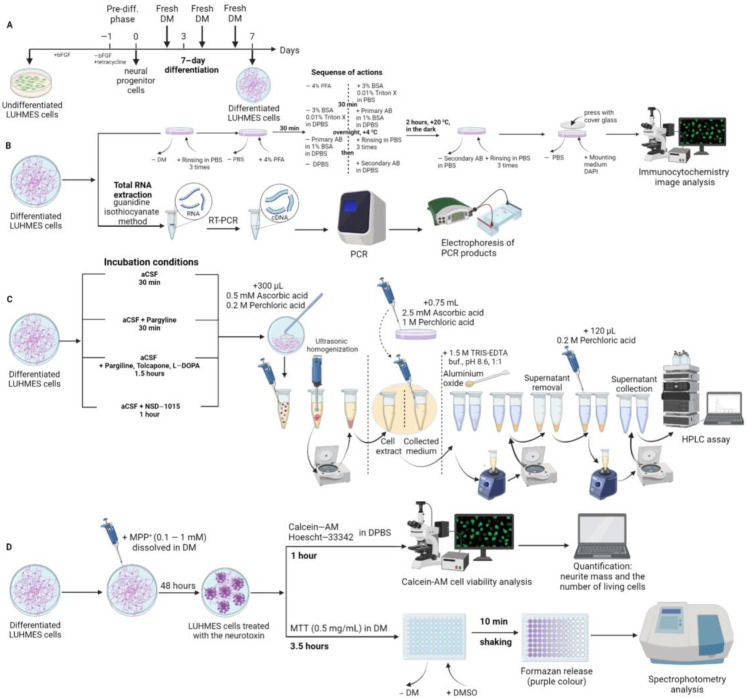
Scheme of LUHMES cell differentiation (**A**), sample preparation and methods used (**B**–**D**). Abbreviations: AB—Antibodies, aCSF—Artificial cerebrospinal fluid, bFGF—Basic fibroblasts growth factor, BSA—Bovine serum albumin, Calcein-AM—Calcein-acetoxymethyl ester; DPBS—Dulbecco’s phosphate-buffered saline, HPLC—High performance liquid chromatography, PFA—Paraformaldehyde, MTT—3-(4,5-dimethylthiazol-2-yl)-2,5-diphenyltetrazolium bromide, MPP^+^—1-methyl-4-phenylpyridinium iodide, “+”—Adding a component to a well/Petri dish, “–“—Removing a component from a well/Petri dish.

The culture medium was removed from the Petri dish with the culture of undifferentiated cells, the cell layer was washed 3 times with 1.0 mL DPBS, the cells were removed from the substrate by pipetting them with a solution of trypsin (0.05%) with EDTA, and after a 1:4 dilution with DMEM/F12, the cell suspension was centrifuged at 200× *g* for 10 min. The cell precipitate was resuspended by pipetting in the pre-differentiation medium (DMEM/F12 supplemented with 1% N-2 supplement, 2 mM Glutamine, 15 mM HEPES (Gibco), and 1 µg/mL tetracycline (Sigma)) and the cells were counted in a hemocytometer. The cells were planted into a specially prepared plastic dish (see paragraph 4.1.1.) with the density required for a particular study: for immunocytochemistry—2–4 × 10^4^/cm^2^ (24-well plate); for HPLC assay—11 × 10^4^/cm^2^ (60 mm Petri dish), for PCR—16 × 10^4^/cm^2^ (35 mm Petri dish), for calcein cell viability test—50 × 10^3^/cm^2^ (48-well plate), for 3-(4,5-dimethylthiazol-2-yl)-2,5-diphenyltetrazolium bromide test—50 × 10^3^/cm^2^ (96-well plate, all plates—Corning, Glendale, AZ, USA) and were cultured at 37 °C in air and 5% CO_2_ for 4 to 12 h, while monitoring the process of cell attachment under a Leica DCF420 C microscope (Leica, Wetzlar, Germany). Non-adherent cells were removed by washing, while adherent cells were placed into the differentiation medium (DMEM/F12 containing 1% N-2 supplement, 2 mM Glutamine, 15 mM HEPES, 1 µg/mL tetracycline, 1 mM dibutyryl 3′,5′-cyclic adenosine monophosphate sodium salt (Sigma), and 2 ng/mL glial cell line-derived neurotrophic factor (Gibco)). The cells were cultured in an incubator at 37 °C in air and 5% CO_2_. The medium was changed every 2 days. Cells were selected for analysis or experiments after 7 days of differentiation (Figure 10A).

#### 4.1.4. Experiments with LUHMES Cells

On the 7th day of differentiation, the differentiation medium was replaced in some cases with the incubation medium (Advanced DMEM/F12) (1:1) with the addition of 1% N-2 supplement, 2 mM glutamine, and 15 mM HEPES, and in other cases, the differentiation medium was replaced with artificial cerebrospinal fluid (NaCl 126 mM, KCl 2.5 mM, CaCl_2_ 2.4 mM, MgCl_2_ 1.2 mM, NaHCO_3_ 25 mM, D-glucose 11 mM, HEPES 20 mM, and ascorbic acid 0.5 mM, pH 7.2). The final volume of the medium in each dish was 3 mL.

In the first series of experiments, LCs were incubated for 30 min—Either in artificial cerebrospinal fluid without any supplements, or in the one containing pargyline hydrochloride at a concentration of 30 μM.

In the second series of experiments, cells were incubated in artificial cerebrospinal fluid: first, 30 min after the addition of 30 μM pargyline hydrochloride, a monoamine oxidase B inhibitor, and 10 μM tolcapone, a catechol-O-methyltransferase inhibitor, and then another 1.5 h after the addition of 100 nM L-DOPA, the immediate precursor of DA.

In the third series of experiments, cells were incubated for 1 h in artificial cerebrospinal fluid containing 500 μM of 3-hydroxybenzylhydrazine dihydrochloride, a specific inhibitor of AADC (all reagents: Sigma).

After the end of the cell incubation according to the above protocols, the incubation medium was collected and HClO_4_ at a final concentration of 0.2 M and ascorbic acid at a final concentration of 0.5 mM were added to it. The samples were frozen in liquid nitrogen and stored at –70 °C until further analysis.

Before cell collection, 0.3 mL of 0.2 M HClO_4_ and 0.5 mM of ascorbic acid were added to the Petri dish. The cells were removed from the Petri dish using a special scraper, and the resulting suspension was transferred to a microtube. After that, the sample was subjected to a single freeze–thaw to destroy the cells. The destroyed cells were homogenized using an ultrasonic homogenizer (UP100H, Hielscher, Teltow, Germany), frozen in nitrogen, and stored at –70 °C until further analysis.

Data on the content of pyridoxal-5-phosphate, L-DOPA, and DA in cells and media were normalized to mg of cell protein. The content of protein in cells was determined by the previously described bicinchoninic acid protein assay method [65]. The sample was taken in a volume of 10 μL after ultrasonic homogenization of cells.

### 4.2. Cell Fixation and Immunostaining

After proliferation of LUHMES stock cells and their subsequent differentiation in 24-well plates (Corning, Glendale, AZ, USA) (Figure 10), immunocytochemistry was performed. To do this, the culture medium was removed, and the cells were washed twice for 5 min in a standard DPBS solution at 20 °C. Next, the cells were fixed for 15 min in 4% paraformaldehyde (Sigma) in 0.1 M phosphate-buffered saline (pH 7.2–7.4) at 20 °C. After that, the fixative was removed, and the fixed cells were washed twice for 10 min in DPBS solution and incubated with 3% bovine serum albumin (Sigma) and 0.1% Triton X-100 (Sigma) in DPBS for 30 min at 20 °C.

The incubation medium containing 3% bovine serum albumin and 0.1% Triton X-100 was changed to a solution of 1% bovine serum albumin and 0.05% Triton X-100 in DPBS containing the primary antibodies in selected dilutions. Cells were incubated with the primary antibodies for 2 h at 20 °C or for 36 h at +4 °C. They were then washed three times for 10 min at 20 °C with DPBS and incubated for 2 h at 20 °C in the dark with the secondary antibodies, the immunoglobulins of the animal that produced the primary antibodies, in DPBS (Table 2).

After incubation with secondary antibodies, the cells were washed twice in the dark with DPBS for 10 min at 20 °C. The cells were then air-dried and placed in hydrophilic embedding medium (Abcam, Cambridge, UK) containing DAPI—Cell nuclear dye.

### 4.3. High Performance Liquid Chromatography

The content of L-DOPA and DA were measured with the use of HPLC with electrochemical detection. The samples were purified from admixtures that hindered the detection of monoamines using the aluminum oxide extraction method. The internal standard dihydroxybenzoic acid at a final concentration of 5 pmol/mL was preliminarily added to the samples. A total of 30–35 mg dry powdered aluminum oxide (Sigma) and chilled 1.5 M TRIS buffered saline with pH = 8.6 (Sigma) were added to each sample in a ratio of 1:1 by volume. For the extraction of dihydroxybenzoic acid, L-DOPA, and DA, the samples were mixed on a rotor in the dark at 4 °C for 10–15 min. Thereafter, the aluminum oxide powder was washed twice with distilled water by centrifuging for 5 min at 4000 rpm and then carefully removing the water. Elution of dihydroxybenzoic acid, L-DOPA, and DA from aluminum oxide was performed by adding 120 μL of 0.2 M HClO_4_. The resulting acid samples with the eluted analytes were analyzed by HPLC (Figure 10).

Separation of dihydroxybenzoic acid, L-DOPA, and DA was carried out on a ReproSil-Pur ODS-3 column, 4 × 100 mm, with a pore diameter of 3 µm (Dr. Majsch GMBH, Ammerbuch, Germany) at +30 °C and a mobile phase velocity from 1 mL/min, supported by an LC-20ADsp liquid chromatograph (Shimadzu, Kyoto, Japan). Mobile phase composition: 1.1 mM 1-octanesulfonic acid sodium salt, 0.1 mM EDTA, and 5% acetonitrile (all reagents from Sigma) in 0.1 M citrate-phosphate buffer (pH 2.58). To measure L-DOPA and DA, a Decade II electrochemical detector (Antec Leyden, Hoorn, The Netherlands), equipped with a glassy carbon working electrode (+0.80 V) and an Ag/AgCl reference electrode, was used. The peaks of the target substances (DA and L-DOPA) were identified by the time of their release in the standard solution. DA, L-DOPA, and dihydroxybenzoic acid standards were used at a concentration of 5 pmol/mL. The content of these substances in the samples was calculated by the internal standard method, using the ratio of the peak areas in the mixture of standards and in the sample, using the LabSolutions software (Shimadzu).

Pyridoxal-5-phosphate was measured using HPLC with an RF-20A fluorescent detector (Shimadzu) at the following wavelengths: λex = 300 nm and λem = 400 nm. Pyridoxal-5-phosphate (Sigma) at a final concentration of 50 pmol/mL was used as an external standard, without extraction on aluminum oxide. Assay conditions were similar to those described above for the measurement of DA and L-DOPA.

### 4.4. Quantitative Real-Time Polymerase Chain Reaction and Agarose Gel Electrophoresis

Real-time PCR was used to assess the expression of the targeted genes: *TH*, *DAT*, *VMAT2*, *AADC*, and housekeeping gene *GAPDH* in undifferentiated and differentiated LCs. Total RNA was isolated with Trizol (ThermoFisher, Waltham, MA, USA) according to the supplier’s protocol. Single-strand cDNA was synthesized from 1 μg total RNA using RevertAid Synthesis Kit (Thermo, USA) according to the supplier’s protocol. Gene expression was estimated by real-time PCR using the Eurogen qPCR Kit (Eurogen, Moscow, Russia). This kit permits you to carry out PCR in 50 cycles. The mRNA level was calculated using the 2^−ΔΔC(t)^ method [66]. Primers used are indicated in Table 3.

After the end of the reaction, all the amplified products were separated at a voltage of 100 V in 1.5% agarose gel (Helicon, Kharkiv, Ukraine) containing ethidium bromide (Thermo Fisher Scientific, Waltham, MA, USA). For additional determination of alternative AADC splice variants, 1.2% agarose gel was used. cDNA fragments were detected using ChemiDoc Touch (Bio-Rad Laboratories, Hercules, CA, USA) and their length was determined against GeneRuler 100 DNA Ladder (Thermo Scientific).

### 4.5. Modeling of Parkinson’s Disease on LUHMES Cells Using 1-methyl-4-phenylpyridinium Iodide

On the 7th day of cultivation, the initial differentiation medium in the wells was replaced: (i) in the first case with the same medium, and (ii) in the second case, with the same medium containing MPP^+^ in the concentration range from 0.1 mM to 0.9 mM (Figure 10).

#### 4.5.1. Cell Survival Assessment

After two days of incubation with MPP^+^, the cells were washed in differentiation medium and incubated for 30 min at +37 °C in DMEM/F12 medium containing 1 µM calcein-AM (Sigma), a dye of living cells, and 1 µg/mL Hoechst-33342 (Sigma), a dye for the nuclei of living and dead cells. Then, the above medium was changed to Hanks’ Balanced Salt solution and the cells were photographed using a Leica DMI 6000 fluorescent microscope (Leica, Wetzlar, Germany) in 2 channels: 365/405 nm for Hoechst-33342 and 475/525 nm for calcein-AM at the same exposure and diode glow intensity. Calcein-AM was used to stain both the cell bodies and the processes (neurites) of neurons. Despite the fact that Hoechst-33342 stains the nuclei of both living and dead cells, the dye penetrates into dead cells faster, so their nuclei glow brighter [67]. Only those cells were considered to be dead in which, despite nucleus staining with Hoechst-33342, cell staining with calcein-AM was not detected. The images obtained by microscopy were used for the semi-quantitative assessment of the “neurite mass” and the quantitative assessment of the number of neurons (cell bodies).

#### 4.5.2. Evaluation of the Functional Activity of Mitochondria Using the 3-(4,5-dimethylthiazol-2-yl)-2,5-diphenyltetrazolium Bromide Test

On the 7th day of cell cultivation in the differentiation medium, it was replaced with the same medium, but with the addition of MPP^+^ at a concentration of 0.1 to 1.0 mM in increments of 0.1 mM. After two days of incubation, the medium was replaced with the differentiation medium with 3-(4,5-dimethylthiazol-2-yl)-2,5-diphenyltetrazolium bromide at a final concentration of 0.5 mg/mL, followed by incubation for 3.5 h at 37 °C. The incubation medium was then carefully removed and 200 μL DMSO was added to the LCs in each well for 10 min incubation at 37 °C. The purple extract was transferred to a 96-well plate. Optical absorbance was measured on a plate reader (Bio-Rad) at 540 nm (reference wavelength 630 nm).

### 4.6. Microscopy, Image Analysis

LC preparations were studied in fluorescence microscopes—Zeiss LSM 880 (Jena, Germany), and Leica DMI 6000 (Wetzlar, Germany). The FiJi software (https://imagej.net/Fiji, accessed on 5 September 2022) was used to assess neuronal loss (death) and neurite degradation. Images of cells stained with calcein-AM were converted into the 8-bit format. After that, the gray threshold was set so that the stained neurites were displayed in grayscale, and the rest of the space remained black. After that, the “Area Fraction” function was used to calculate the area of the well surface covered by the neurites. The number of cells was counted by converting images from Hoechst 33,342 into the 8-bit format. Next, we set the gray threshold in such a way that only the nuclei were colored, and the background remained black. The “Watershed” function was then used to separate the nuclei which were “stuck together”. After that, the function “Analyze Particles” was used to count the cells.

Quantitative analysis of TH-, AADC-, and DAT-immunopositive differentiated LCs was carried out according to a previously described method [68]. LCs were photographed in each well at 20× magnification in randomly selected fields of 0.14 mm^2^. Then, in each photograph the total number of LCs was counted by the number of nuclei stained with DAPI, as well as the number of LCs immunopositive for TH, AADC and DAT.

### 4.7. Statistical Analysis

The obtained data were statistically processed using GraphPad Prism 8.0 software (GraphPad Software, San Diego, CA, USA). For a statistical comparison of the control and experimental groups, the Student’s *t*-test was used. The data are presented as mean ± standard error of the mean (SEM). A *p* value < 0.05 was considered to be statistically significant.

## 5. Conclusions

This study has attempted to advance the fight against neurodegenerative diseases, specifically, PD. To this end, we have developed a cellular model of a key link in the pathogenesis of PD: the degeneration of nigrostriatal DAergic neurons involved in the regulation of motor function. The undoubted advantage of this model is the fact that it is based on the use of human nigral DAergic neurons (differentiated LCs). It is also important to note that obtaining differentiated DAergic neurons from LCs is a fast, easily reproducible, and cheap procedure. Using a comprehensive methodological approach—Immunocytochemistry with image analysis, real-time PCR, and HPLC, it was shown that all cultured LCs after differentiation express not only a neuronal, but also a DAergic phenotype. This made it possible to further develop a PD model using MPP^+^, a neurotoxin which is captured into DAergic neurons by the DAT. This model was tested using specially selected structural and functional markers of the progressive degradation of DAergic neurons. The development of this PD model opens up broad prospects for studying some of the molecular mechanisms of PD pathogenesis and for testing antiparkinsonian drugs.

## Figures and Tables

**Table 1:** Main characteristics of the dopaminergic phenotype of differentiated LUHMES cells, UKN and ATCC cell lines, according to the previous and this study.

Characteristic of DAergic Phenotype	UKN Line (Literature Data)	ATCC Line (Literature Data)	ATCC Line (Our Data)
TH	mRNA: + + [32] Protein: + + [28,29]	mRNA: + + [32] Protein: + + [10]; +/− [32,42]	mRNA: + + Protein: + +
AADC	mRNA: + + [29] Protein: − −	mRNA: + + [10] Protein: − −	mRNA: + + Protein: +
DAT	mRNA: + + [32] Protein: + [32].	mRNA: + + [32] Protein: + [32]	mRNA: + + Protein: + +
VMAT2	mRNA: + + [32] Protein: + + [43,44]	mRNA: + + [32] Protein: − −	mRNA: + + Protein: + +
DA	+ + [28,29]	+ − [32]	+ −

+ +, easily detected; +, poorly detected; -, not detected; - -, not studied. Gene expression was assessed by PCR real time, and proteins were determined using Western blot or immunocytochemistry. Abbreviations: ATCC—American Type Culture Collection, AADC—Aromatic L-amino acid decarboxylase, DA—Dopamine, DAergic—Dopaminergic, DAT—Dopamine transporter; mRNA—Messenger ribonucleic acid; TH—Tyrosine hydroxylase, VMAT2—A vesicular membrane transporter type 2, UKN—University of Konstanz, original provider.

**Table 2:** Primary and secondary antibodies used for immunostaining of differentiated LUHMES cells.

Type of Antibodies	Manufacturer/Cat. No.	Specification	Dilution
Primary	Abcam (Cambridge, UK), ab111468	Rabbit anti-DAT, polyclonal	1:100
Primary	Abcam (Cambridge, UK), ab32454	Rabbit anti-MAP2, polyclonal	1:250
Primary	SySy (Goettingen, Germany), 105011	Mouse anti-Synaptotagmin 1, monoclonal	1:200
Primary	Sigma (St. Louis, MO, USA), AB1598P	Rabbit anti-VMAT2, polyclonal	1:75
Primary	R&D systems (Minneapolis, MN, USA), AF3564	Goat anti-AADC, polyclonal	1:80
Primary	Santa Cruz (Dallas, TX, USA), sc-7011-R	Rabbit anti-α-Synuclein, polyclonal	1:250
Primary	Abcam (Cambridge, UK), ab7751	Mouse anti-β-Tubulin III, monoclonal	1:1500
Primary	Invitrogen (Waltham, MA, USA), PA5-37740	Rabbit anti-Phospho-α-Synuclein (Ser-129), polyclonal	1:750
Primary	Thermo Fisher (Waltham, MA, USA), 701949	Rabbit anti-TH, polyclonal	1:250
Primary	Sigma (St. Louis, MO, USA), T1299	Mouse anti-TH, monoclonal	1:500
Secondary	Invitrogen (Waltham, MA, USA), A10040	Donkey anti-Rabbit, Alexa Fluor 546	1:700
Secondary	Invitrogen (Waltham, MA, USA), A32766	Donkey anti-Mouse, Alexa Fluor 488	1:1000
Secondary	Invitrogen (Waltham, MA, USA), A21469	Chicken anti-Goat, Alexa Fluor 647	1:1000

Abbreviations: AADC—Aromatic L-amino acid decarboxylase. DAT—Dopamine transporter, MAP2—Microtubule-associated protein 2, TH—Tyrosine hydroxylase, VMAT2—Vesicular monoamine transporter 2.

**Table 3:** The nucleotide primers used in this study.

Gene Name	Forward Primer	Reverse Primer
TH	CGGGCTGTCCTCCTA	AGCTTGTCCTTGGCGTCACT
DAT	CAGCCTGCAGACCACACTC	AGGCTCGCGGATACTGC
VMAT2	CCGGGAATGCTACCAGAGAC	CGAGGCAAACAACAGACCAA
AADC	GGCTTCCCATGTCTTTCTCC	GGCTTTCCTACCACTTCTGATG
AADC to test alternative splicing	GCCACCAGCTTCTCCATGA (516)	AGAGGGAAGGAGATGGTGGATTA (719)
GAPDH	GGGGGAGCCAAAAGGGTCATCATCT	GAGGGGCCATCCACAGTCTTC

Abbreviations: AADC—Aromatic L-amino acid decarboxylase. DAT—Dopamine transporter, GAPDH—Glyceraldehyde 3-phosphate dehydrogenase (housekeeping gene), TH—Tyrosine hydroxylase, VMAT2—Vesicular monoamine transporter 2.

## Data Availability

The data presented in this study are available on request from the corresponding author. The data are not publicly available due to legal issues.

## Supplementary Materials

The following supporting information can be downloaded at: https://www.mdpi.com/article/10.3390/ijms24010733/s1.

## Abbreviations

AADC	aromatic L-amino acid decarboxylase
ATCC	American Type Culture Collection
DA	dopamine
DAergic	dopaminergic
DAPI	4′,6-diamidino-2-phenylindole dihydrochloride
DAT	dopamine transporter
DPBS	Dulbecco’s phosphate-buffered saline without Ca^2+^ and Mg^2+^
LCs	LUHMES cells
L-DOPA	L-3,4-dihydroxyphenylalanine
MAP2	microtubule-associated protein 2
MPP^+^	1-methyl-4-phenylpyridinium iodide
PCR	polymerase chain reaction
PD	Parkinson’s disease
TH	tyrosine hydroxylase
UKN	University of Konstanz
VMAT2	vesicular monoamine transporter 2
