# Supplementary material for: LUHMES Cells: Phenotype Refinement and Development of an MPP+-Based Test System for Screening Antiparkinsonian Drugs
Source: Int J Mol Sci. 2023 Jan 1;24(1):733. doi: 10.3390/ijms24010733 (PMC9821222; doi:10.3390/ijms24010733)
Supplement: Supplementary file 1 [file ijms-24-00733-s001.zip › ijms-2026612-supplementary.pdf]

Expression of dopaminergic markers in LUHMES cells on day 7 of differentiation and in undifferentiated cells

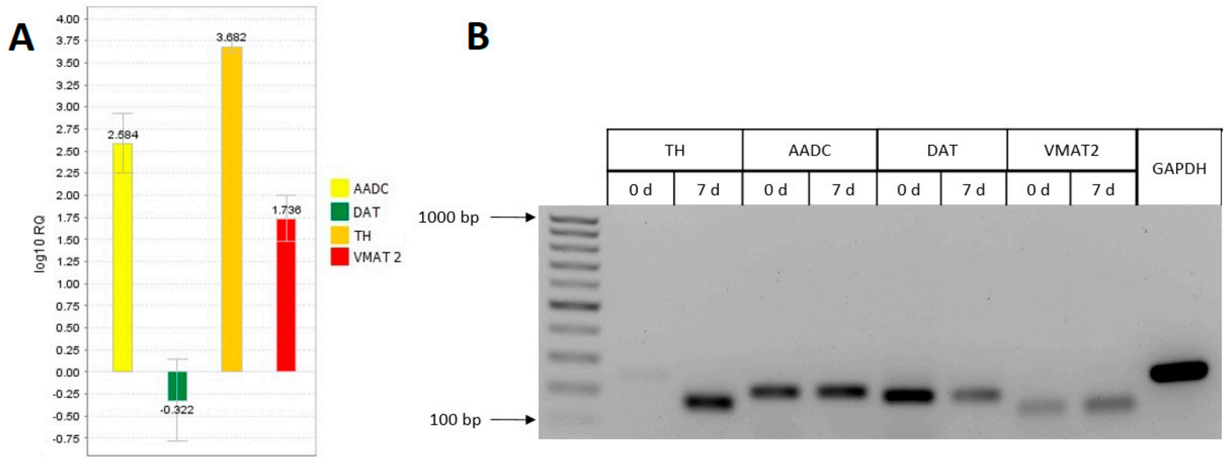

Figure S1. (A) Changes in the expression of genes of the dopaminergic phenotype in LCs on day 7 of differentiation compared to undifferentiated LC cells (qPCR, normalized to GAPDH). (B) - Agarose phoresis of PCR products of dopaminergic phenotype genes in undifferentiated and differentiated (day 7 of differentiation) LCs. A DNA ladder of 100+ bp was used. (Evrogen, Russia).

In differentiated cells, the expression of all protein genes, apart from DAT, increased.

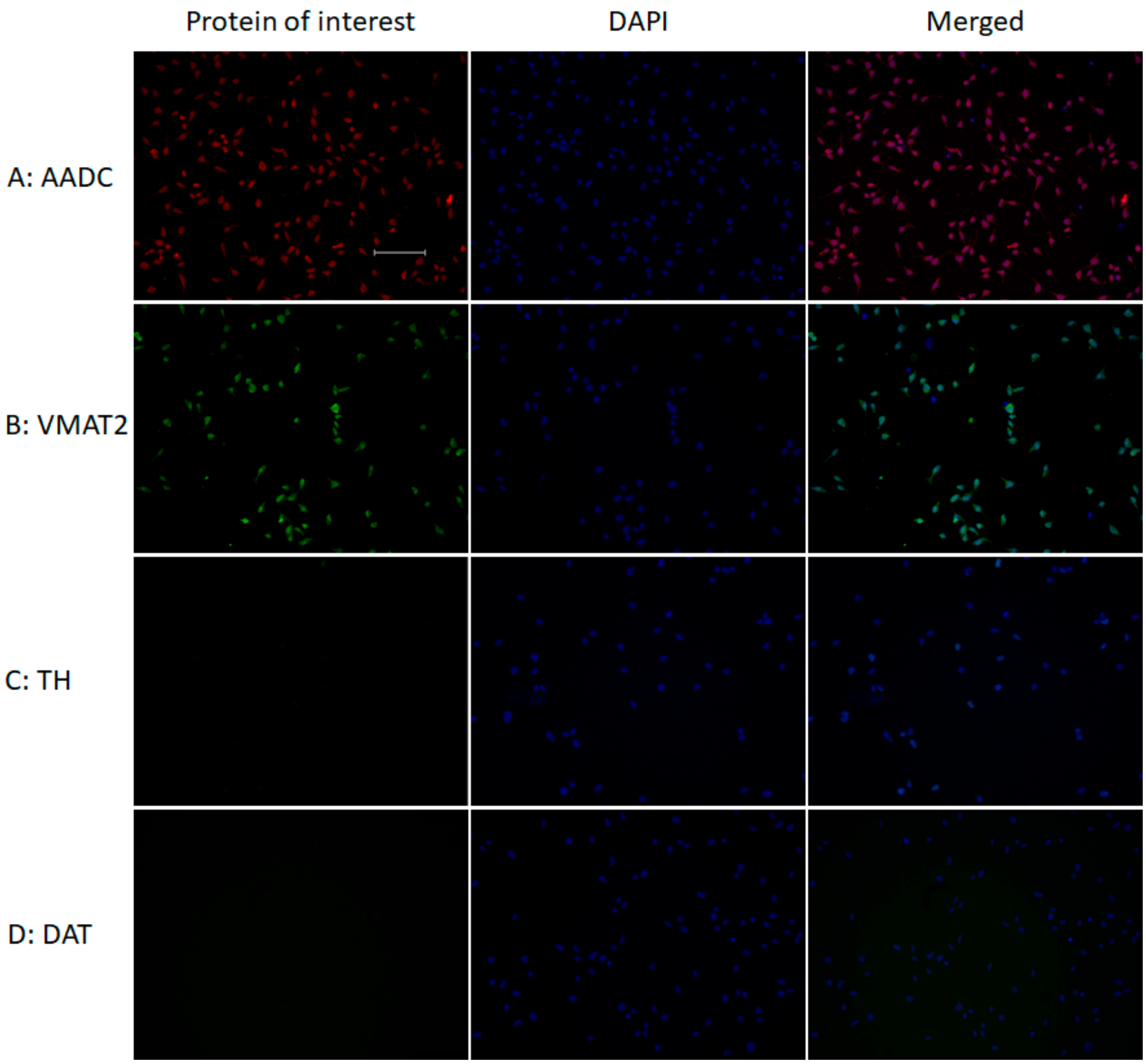

Figure S2. Immunostaining of undifferentiated LCs for proteins of the dopaminergic phenotype: immunopositive cells for AADC and VMAT2 (A, B) and immunonegative for TH and DAT (C, D).

In the immunocytochemical study of undifferentiated LCs, we detected the expression of AADC and VMAT2, but did not detect the expression of TH and DAT.

Pyridoxal-5-phosphate content in differentiated LUHMES cells

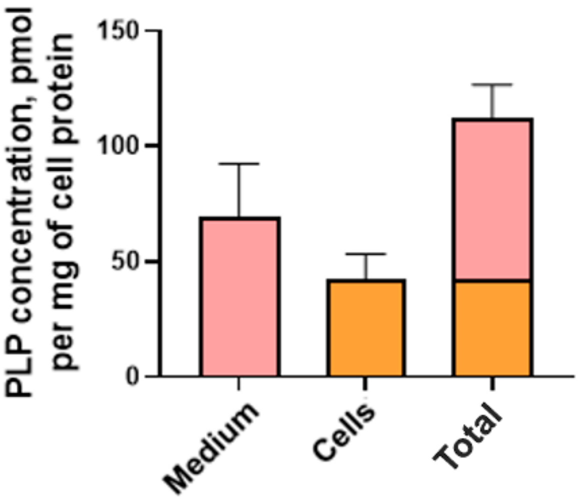

Figure S3. The concentration of pyridoxal 5'-phosphate (PLP) in cells and artificial cerebrospinal fluid. Samples were collected in 30 minutes after replacement of differentiation medium with artificial cerebrospinal fluid.
